# Key Informant Interviews to Inform Nutrition and Physical Activity Recovery Efforts in Child Care Settings amid the COVID-19 Pandemic in the United States

**DOI:** 10.3389/fpubh.2022.888368

**Published:** 2022-06-14

**Authors:** Allison M. Nitto, David Berrigan, Andrew A. Bremer, Sarah K. Kersten, Leah R. Carpenter, Amy L. Yaroch

**Affiliations:** ^1^Gretchen Swanson Center for Nutrition, Omaha, NE, United States; ^2^Health Behaviors Research Branch, Behavioral Research Program, Division of Cancer Control and Population Sciences, National Cancer Institute, National Institutes of Health, Bethesda, MD, United States; ^3^Eunice Kennedy Shriver National Institute of Child Health and Human Development, National Institutes of Health, Bethesda, MD, United States

**Keywords:** child care, pandemic recovery, COVID-19, nutrition, physical activity, early childhood education

## Abstract

**Purpose:**

The COVID-19 pandemic created a series of challenges for children's health, including several challenges related to nutrition and physical activity in child care settings. Thus, this study explored: 1) how COVID-19 impacted nutrition and physical activity in child care settings and how to address these challenges moving forward; 2) potential best practices in child care that emerged during the COVID-19 pandemic worth continuing; and 3) future directions for accessing, implementing, and evaluating COVID-19 federal investments in child care settings.

**Methods:**

The study utilized a qualitative content analysis approach. In June 2021, the investigators conducted 17 qualitative interviews with federal representatives (*n* = 4), practitioners (*n* = 7), and researchers (*n* = 6). Recruitment continued until saturation was achieved. Virtual interviews lasted approximately 45 to 60 minutes and were recorded, transcribed, and coded for themes and subthemes using thematic content analysis.

**Results:**

Primary findings included: 1) COVID-19 likely increased stress and exacerbated prevalence of food insecurity for child care staff and participating families, and decreased diet quality among children both while in and outside of child care; 2) flexibilities to federal child care requirements, outdoor learning opportunities, and meal provision strategies implemented during the pandemic were perceived as positive and could continue post-pandemic; and 3) future efforts could utilize the recovery funds to make changes that are equitable and sustainable, such as conducting equity assessments and collaborating with community organizations, along with evaluating impacts of these efforts on food insecurity and health of child care staff and participating children.

**Conclusion:**

Overall, recommendations focused on several social determinants of health, including addressing food insecurity among both children and staff, and infrastructure for nutrition and physical activity. Continued programmatic and public health recovery efforts aimed to mitigate the negative impacts of COVID-19 are critical to fostering health and wellbeing in child care settings.

## Introduction

The child care system is a critical component to aid in child development in the United States (U.S.). In 2019, an estimated 59 percent of children five and younger were in non-parental care at least 1 day a week, and of those, 82 percent were enrolled in center-based care or family day care homes (1). Child care quality and access is a key area of the social determinants of health (SDOH) which affect a wide range of health risks and outcomes (2). Notably, research has shown that the child care system plays a critical role in childhood health and wellness, including the prevention of obesity (3). The Coronavirus Disease 2019 (COVID-19) pandemic devastated many systems in the U.S., including child care settings (4). A recent study estimated that preschool enrollment decreased 14 percentage points from 2019 to 2020 (5). Research shows that the pandemic resulted in lockdowns, which reduced nutritional quality of children's diets, food security status, and physical activity levels while children were not in child care (4, 6, 7).

COVID-19 response and recovery efforts in child care are likely to have short-term and longer-term impacts on behavioral, household, societal, and economic influences on population health and well-being, including weight status (8). In 2020, the National Collaborative on Childhood Obesity Research (NCCOR) – a public-private partnership among the National Institutes of Health (NIH), the Centers for Disease Control and Prevention (CDC), the Robert Wood Johnson Foundation, and the U.S. Department of Agriculture (USDA) – formed a scientific workgroup to examine the impact of COVID-19 and the subsequent COVID-19 federal recovery investments (e.g., Coronavirus Aid, Relief, and Economic Security Act (CARES) and American Rescue Plan Act (ARPA)) on child care systems and child well-being.

CARES, enacted on March 27, 2020, included $3.5 billion for the Child Care and Development Block Grant and $750 million for Head Start programs (9) CARES also issued guidance that allowed states more flexibility in meeting Child Care and Development Fund (CCDF) requirements by adapting policies to maintain services for families affected by COVID-19. For instance, flexibilities include: continue to pay child care providers who accept subsidies during closure or low attendance; provide emergency care through temporary regulatory changes; provide additional funding to providers who offer care for children of essential workers; and waive or cover a portion of child care tuition that families may otherwise be required to pay (9, 10) The ARPA was signed into law on March 11, 2021 and provided $39 billion for the child care industry, including $24 billion for a child care stabilization fund and $15 billion for the CCDF (10). These funds help child care providers “to reopen or stay open, provide safe and healthy learning environments, keep workers on payroll, and provide mental health supports for educators and children” (10). These funds are critical to support non-health care frontline workers, like child care workers, considering emerging research has found child care staff experienced moderate stress and were more likely to experience symptoms of depression (11). The plan also “provides more flexible funding for states to make child care more affordable for more families, increase access to high-quality care for families receiving subsidies, increase compensation for early childhood workers, and meet other care needs in their states” (10).

The rapid infusion of large investments in child care infrastructure via the CARES, ARPA, and other recovery bills has the potential to meaningfully influence SDOH and child nutrition and physical activity outcomes. Thus, research on the impacts of COVID-19 and the utilization of the CARES and ARPA funds in child care is critical. A qualitative study design was employed to gather insights from federal agency representatives, child care practitioners, and public health researchers on: 1) how COVID-19 affected nutrition and physical activity in child care settings and how to address challenges resulting from the pandemic; 2) adjustments in child care settings that emerged throughout the pandemic as potential best practices worth continuing; and 3) future directions for accessing, implementing, and evaluating the CARES and ARPA child care investments.

## Methods

### Design

The study utilized a qualitative content analysis approach. In June 2021, 17 in-depth, semi-structured interviews were conducted with key informants among three subgroups: federal representatives (*n* = 4), child care practitioners (*n* = 7), and public health researchers (*n* = 6). Most interviews were conducted with one key informant; however, there were two instances of two participants joining together in one interview (one federal representative and one practitioner interview). Purposeful sampling was used to identify potential interviewees with experience and expertise in child care policies, practices, and research (12). The investigators worked with NCCOR representatives to identify potential interviewees with expertise in child care. The investigators recruited interviewees via email. In some cases, proposed interviewees recommended alternative and/or additional representatives from their organizations or those they knew working in the area. Recruitment continued until saturation was reached. Saturation was defined as the point where no new data is being collected and the data provided is redundant of what was collected during prior interviews (13, 14). Therefore, the investigators reviewed the transcripts and met weekly to determine if new data emerged during the interviews. The investigators also ensured saturation was achieved across the three interviewee subgroups by checking for no new data and redundant data for each of the specific questions asked of each interviewee type. After approximately 3 weeks and 17 interviews, the investigators determined that saturation occurred, and thus, no additional interviews were needed. The study protocol was approved by the University of Nebraska Medical Center's Institutional Review Board.

### Interviewee Characteristics

The interviewees represented 15 different organizations located across the United States. Among researchers, areas of expertise included food insecurity, nutrition security, health disparities, physical activity, and child care as it relates to federal nutrition programs (e.g., Child and Adult Care Food Program (CACFP), Special Supplemental Nutrition Program for Women, Infants, and Children (WIC), and Supplemental Nutrition Assistance Program (SNAP)). Their work primarily centered around young children and families, specifically low-income families. Researchers provided specific feedback on future directions for evaluating child care utilization of CARES and ARPA funding. Practitioners contributed perspectives from both the non-profit and for-profit sectors, commercial centers and family day care homes, and at the national and state levels. Their involvement in child care spanned programmatic, policy, and advocacy duties, within the topic areas of nutrition, physical activity, wellness, food systems, and federal nutrition programs. Federal representatives worked across different agencies, such as the Administration of Children and Families' Office of Child Care and Early Child Development and Office of Minority Health, and described their work in child care settings related to nutrition and physical activity priorities. They reported that responsibilities included but were not limited to overseeing health services for Head Start, crafting policy, training, and technical assistance for child care staff, and building systems within child care.

### Data Collection

A semi-structured interview guide included questions asked of all interviewees along with tailored questions and probes specific to each of the three interviewee groups to align with their specific area of expertise. The core interview questions focused on identifying the impacts of COVID-19 on childhood nutrition and physical activity, the nutrition and physical activity opportunities that emerged due to COVID-19, and how recent CARES and ARPA investments may affect child care settings, as well as how these investments could be used to strengthen nutrition and physical activity in these settings. Table 1 includes the core interview questions. In addition, probing was used to gather more details through non-verbal pauses and verbal follow-up questions, as needed. The interview guide was reviewed by the NCCOR workgroup, which consisted of staff from NIH, CDC, and FHI 360 (a non-profit human development organization and the managing organization for NCCOR). The guide was then updated based on NCCOR workgroup feedback. Key changes included streamlining the questions, clarifying terms, and changing the order of the questions.

**Table 1:** Semi-structured interview guide.

1. Can you tell me a little bit about yourself and your current work, especially as it is related to childhood nutrition and physical activity, if applicable?
2. Has your current research explored the impacts of COVID-19 on childhood nutrition and physical activity?
3. Thinking about nutrition and physical activity strategies, policies, and practices in child care programs, how have they changed since the COVID-19 pandemic? What nutrition and physical activity opportunities emerged due to COVID-19?
4. What do you think recovery will look like for these programs?
5. Can you talk about some potential natural experiments that can be developed to evaluate aspects of the COVID-19 recovery legislation related to early childhood?
6. How do you think these investments will affect the child care landscape overall and among programs that serve children and families disproportionately impacted by COVID-19?
7. What is needed to support effective implementation of the legislation?
8. What is needed to increase the urgency/priority of implementing these strategies in child care programs?
9. What is needed to support effective maintenance of the legislation long-term?

*Core questions asked during semi-structured interviews*.

Three investigators trained in qualitative interviewing conducted the interviews. Two of the investigators held master's in public health (MPH) degrees and the other investigator held a doctorate degree in nutritional sciences. All three investigators had recent experience collecting and analyzing qualitative data along with the expertise in child care and child health and wellness. The interviews were conducted by telephone or Zoom video conference. The investigators had no conflict of interest or other relationship with the interviewees that could influence the data collected. Each interview took approximately 45–60 min to complete. Participants were informed that the interview was voluntary, and they had the option to end the call at any time or choose not to answer a question for whatever reason. However, none of the interviewees refused or ended the call early. Interviews were audio recorded with participants' permission and transcribed verbatim by a professional transcription service. Participants were provided a $30 incentive in the form of a gift card for those who could accept it.

### Analysis

The qualitative thematic analysis (15) started with the three trained qualitative investigators meeting to review the process for reviewing and coding the qualitative data. The process included all three investigators reviewing all transcriptions to create an initial list of codes based on the study evaluation questions. The investigators also relistened to audio files to resolve any questions regarding the transcripts. Any data identified by the investigators to be unreliable or inaccurate was removed from the coding process. The investigators reconvened to determine the initial coding list and data to include in the analysis. Investigators used NVivo 11 (16) software to organize and code all interview data. The codes were further refined using an iterative process after the first round of coding was completed. The three investigators met several times to triangulate findings and reconcile any differences in coding application. The process also included two additional rounds of coding conducted by the three investigators. This process led to the development of the major themes (*n* = 10) and subthemes (*n* = 7) that are presented in the following findings section. As part the analysis and to ensure feedback from the various interviewee subgroups were represented, the investigators tracked which themes were supported by feedback from the different interviewee groups. Example quotes also are included below to support the themes.

## Results

### Impacts of COVID-19 on Child Care Settings and Potential Solutions

#### Stress on Child Care Staff and Participating Families

Interviewees across all groups reported that child care professionals experienced high levels of both mental and physical stress due to the COVID-19 pandemic and that “stress” was one of the priority issues to address. They described that state and local COVID-19 restrictions caused most child care programs to temporarily close or operate with limited capacity. Practitioners and researchers cited limited staffing as a major challenge to reopening due to perceived health concerns and low wages of child care staff. Researchers noted that child care staff were overwhelmed with implementing new health and safety procedures and needed additional support. One solution mentioned by multiple researchers was to provide child care staff health and wellness support, including mental health support.

The COVID-19 pandemic also caused a high-level of stress among children and families. Practitioners explained that families were hesitant to put their child (ren) back into child care, especially when health and safety plans were in flux. In addition, practitioners indicated that child care closures resulted in disruption to children's daily routine and structure, resulting in child mental health and behavioral issues that were evident once children returned to child care. Interviewees across all groups described that child care staff will need to allocate additional time and resources to helping families transition back into child care. Federal representatives described an increased need for attention and funding at the state level, such as a train-the-trainer model to support child care staff in addressing child mental health issues.

#### Food Insecurity

Interviewees across all groups overwhelmingly indicated that food insecurity was prevalent among families with young children and that food insecurity increased in this group due to COVID-19. Interviewees expressed concern that children did not receive adequate meals and snacks while not attending child care programs. Practitioners and researchers described child care staff as oftentimes being food insecure themselves and reliant on federal food assistance programs and food banks/pantries. Practitioners and researchers described this as problematic, indicating the need to better support child care providers financially. Despite this, interviewees across all groups mentioned that COVID-19 has resulted in an increased national awareness of food insecurity issues in the U.S. This presents an opportunity to create sustainable changes to help mitigate food insecurity among child care staff and the children and families they serve.

#### Nutrition and Physical Activity

Interviewees across all three groups expressed concern regarding the nutritional quality of meals among children not attending in-person child care. One federal representative mentioned that fruits and vegetables may need to be reintroduced to children in the child care setting since some families may not have had access to fruits and vegetables while not attending child care programs during the pandemic. The reintroduction of fruits and vegetables could help increase the likelihood that children will try and consume these healthy foods once returning to child care.

For child care programs that were open, practitioners noted that the nutritional quality of foods served may have deteriorated. Practitioners and researchers described that COVID-19 halted family style dining practices due to health and safety guidelines. Without family style dining, children missed out on critical developmental opportunities, such as gross motor development. However, several practitioners and researchers noted that child care programs were hesitant to reinstate family style dining due to health concerns and that programs may need guidance in doing so. Aside from family style dining, interviewees reported that other nutrition promoting efforts, like taste testing and new recipe development, were reduced due to additional staff time spent on health and safety procedures. In addition, a practitioner also highlighted an increased reliance on processed foods among child care programs that remained opened during the pandemic.

Lastly, interviewees across all groups hypothesized a general reduction of physical activity when children were not in child care. Several practitioners posited that physical activity levels are influenced by the communities' children live in. Lower-income and/or urban neighborhoods may allow for fewer physical activity opportunities due to limited access to outdoor recreation spaces and safety concerns. Interviewees across all groups felt that targeted outreach efforts and strategic planning are needed to bring low-income and ethnic minority community members into conversations with local and state officials within their communities and neighborhoods.

A summary of the themes with supplemental quotes on the impact of COVID-19 child care settings is shown in Table 2.

**Table 2:** Key informant themes and quote snapshot: impacts of COVID-19 on child care settings.

**Theme**	**Example quote**
Increased stress	“*…it was a big change for adults and children, so we've been spending a lot of time focusing on social emotional development efforts, like, how do we help families? How do we help parents? Helping parents and even talking to staff, now that children are coming back into programs, …helping them to understand that the transition isn't going to be that smooth, that children will have behavioral issues [Federal Representative].”*
	“*I think the teachers need, and the directors probably need, some mental health support. COVID was extremely difficult for everybody in that regard. And a lot of the burden was on these essential workers who, I would consider child care [staff] essential workers [Researcher].”*
Increased food insecurity	“*I hear people talking more about it as opposed to us always trying to... or, at least me, always trying to bring up and say, “You know what, remember food insecurity is a problem in our communities. A lot of our families live in food deserts. A lot of our families don't have access to produce that's fresh [Federal Representative].”*
	“*I really do think that the concept of food insecurity became more real for people. I mean, it's been a problem, but because of COVID it became greater... So, all of a sudden there was a lot more people that were dealing with food insecurity. And we saw, in the news, the long lines of cars, and all the places … and people that were just in line and needing to get food. [Federal Representative].”*
Decreased physical activity and nutrition	“*...nutrition just took a nosedive during COVID... I'm sure food preparation from a cook's perspective would also include more reliance on processed things. Because they didn't have to make it, or just have to keep things safe [Practitioner].”*
	“*Kids aren't outside, right? And we have families and communities where it's not safe to be outside. And so, the public health message of get outside… it's not a reality for a lot of our families to achieve. I think we're going to have a lot of kids who were in front of the TV for long periods of time... [Federal Representative].”*

### Opportunities That Emerged During COVID-19

#### Flexibilities to USDA Requirements

During the COVID-19 pandemic, the USDA issued CACFP waivers that allowed programs flexibility in targeted meal patterns, nutritional requirements, and increased reimbursement rates (17). CACFP waivers were perceived by interviewees across all groups as critical to continue serving children nutritious meals. Practitioners also described that these USDA flexibilities helped stabilize child care programs during the pandemic since the increased reimbursement rates served as a reliable source of income, especially for child care programs that experienced significant drops in enrollment. Prior to COVID-19, meals served at family day care homes (private residences that provide care, meals, and snacks to nonresident children) were either reimbursed at a high (Tier I) or low (Tier II) rate depending on the location of the day care home in a low-income area or the provider's own household income level (18). A few practitioners described that with USDA flexibilities, all family day care programs were allocated the highest reimbursement rate as compared to the tiered eligibility approach used prior to the pandemic. One practitioner recommended that family day care programs continue to receive the highest reimbursement rate to treat all family day care programs equally. Due to health and safety guidelines, as well as CACFP waivers, monitoring was conducted virtually. Practitioners described virtual site visits as more effective and efficient and recommended using a virtual and in-person hybrid model post pandemic.

#### Outdoor Play and Learning

Due to health and safety concerns, interviewees reported that child care settings increased outdoor activities during the COVID-19 pandemic. Practitioners and researchers highlighted the positive impact that outdoor play and learning had on physical activity and mental health of both children and staff. They also recommended shifting additional learning activities to the outdoors post pandemic. Practitioners cited “farm to early care and education (ECE)” (e.g., onsite vegetable gardens) as an opportunity for outdoor learning. They further described that “farm to ECE” provides nutrition education, experiential learning, and connections with produce growing processes and practices, while also allowing for social distancing. A few practitioners and federal representatives suggested maximizing excitement among children once they return to child care settings and reunite with friends in person by supporting outdoor group activities, such as learning trails, center/school gardens, and other activities that can positively impact nutrition and physical activity.

#### Provision of Meals to Families and Children

Interviewees discussed various strategies to serve meals to children and families outside of the child care setting to address food insecurity. Examples included grab and go meals, partnerships with local entities (e.g., grocery stores, restaurants), meal kits, meals in the mail, backpack programs, on-site food pantries, and meal transportation for families. Practitioners noted that these efforts increased access to healthful meals, but gaps in providing meals to eligible children may still exist, such as child care programs that were not able to make contact with certain children and child care programs that do not have the capacity to distribute take home meals. Programs could continue to work with community partners to help with reach and sustainability.

A summary of the themes with supplemental quotes on the opportunities that emerged during COVID-19 is shown in Table 3.

**Table 3:** Key informant themes and quote snapshot: opportunities that emerged during COVID-19.

**Theme**	**Example quote**
Federal flexibilities as critical components	“*It's not as if the program was this huge moneymaker for them [family day care providers]. We're talking simply about having some more equity in the program so that the way that they're reimbursed is on par with child care centers [Practitioner].”*
	“*Well, the federal programs have allowed more flexibility for us to be responsive to the pandemic, especially during its initial few months to have food that was not congregant…as well as being able to request waivers when the nutrition meal plan could not be met. And, again, a lot of those waivers were very pertinent to the peak of the pandemic. [Practitioner]”*
Increased outdoor activities	“*They [child care providers] were basically moving everything outside, and they were just doing stuff outside. So, the outdoors became their learning space...But I think that that's just a plus side of COVID. Because the kids were moving [Researcher].”*
	“*I think a lot of programs have really embraced outdoor education in a way that they just didn't before... That's something we've seen across the country is that outdoor education and the physical activity that kids can get outdoors, I think that we're going to see more outdoor education and a need for higher quality and for training on how to do great high quality outdoor education moving forward [Researcher]*.
Increased efforts addressing food insecurity	“*And so, we knew those children were at home, not necessarily having access to healthy meals or any meals, depending on the family's situation. So, since centers being able to prepare meals in a way that those families could take them home, helped us feel like, ‘Oh, okay, at least the children are getting the meals that they would have been getting if they were in care, until a situation worked out' [Practitioner].”*
	“*I think a big opportunity is elevating the role of ECE and addressing childhood and family food security. We saw what happened when children and families did not have access to those meals provided through early care and education programs. And I think that just reinforces the opportunity of early care and education to be that hub for nutrition resources, for families and the opportunity for it to not just be about access to food, to be access to quality and access to that connection to their community through [the] system. [Practitioner].”*

### Future Directions to Help Recover From COVID-19

#### Staff Support and Training

Several interviewees across all groups indicated that a key component to recovering from the negative impacts of COVID-19 is recruiting and retaining sufficient child care staff, who are mostly negatively impacted by low wages. Interviewees stressed that child care staff salaries need to be increased and sufficient breaks and paid time off need to be available for staff to help with staff satisfaction and retention. *One* federal representative also suggested that CARES and ARPA funds could be used to hire designated nutrition and wellness staff and/or funds be used to incorporate nutrition and wellness training as part of the onboarding training curriculum. This investment could help further empower staff to be “healthy lifestyle” role models for the children participating in their programs.

#### Technical Assistance and Community Partnerships

A few practitioners and federal representatives indicated the potential benefit of an advisory board composed of key partners from the local to federal levels who could support child care programs in utilizing CARES and ARPA funds. Suggested partners included nutrition and physical activity experts and researchers who could advise on child care programs and how to invest money in nutrition and physical activity related items. Community-based organizations also could help ensure funds are being allocated equitably, including social service organizations. Advisory boards could include representatives from federal food assistance programs to help address food insecurity issues. State, regional, and/or federal partners could provide technical assistance to ensure funds are spent in accordance with state and federal laws and regulations. It was also recommended that child care staff and families be involved in various processes to ensure changes made are appropriate and effective for the intended end-users.

#### Sustainability

Multiple interviewees across the three groups indicated that child care programs should use COVID-19 recovery funds to make long-term changes rather than using funds as a short-term “band aid” solution. Sustainable investments related to nutrition and physical activity cited by the interviewees included developing and maintaining: school vegetable gardens, nature trails, nutrition education curriculum, food service equipment, cooking classes, nutrition and physical activity training for staff, technology software, and coordinated, cross-program data sharing systems.

Multiple interviewees across the three groups also suggested that sustained funding is critical for sustainability of child care programs, which could entail embedding child care program funding with other existing federal or state funding and/or taxation earmarked for child care. Researchers and federal representatives indicated that state legislators need to be responsible for identifying ways to ensure child care programs have sufficient and sustainable funding.

#### Equity

Interviewees were asked about suggestions for recovery options specific to programs and families disproportionately impacted by the COVID-19 pandemic. One practitioner recommended ensuring technical assistance and guidance are made available to programs and families impacted the most. This would help maximize awareness of the resources available. One researcher and one practitioner also suggested that COVID-19 recovery funds could be used to invest in local municipalities and community organizations to support those disproportionately impacted by COVID-19. These organizations are often aware of the programs and families most impacted by disaster situations, such as COVID-19, and could connect them with resources to help recover. Noted examples of community organizations included: YMCA, parks and recreation departments, 4H clubs, and local colleges and universities.

One practitioner and one federal representative also suggested that an equity assessment be conducted to help guide those decisions prior to allocating or spending any funds. The assessment could help identify needs and proposed services to address those needs. The equity assessment also could help avoid creating unintended consequences and negative impacts. Child care programs might not have the capacity to do such assessments; therefore, other partners at the state and local levels could potentially help.

#### Research Needs

**Figure 1:**
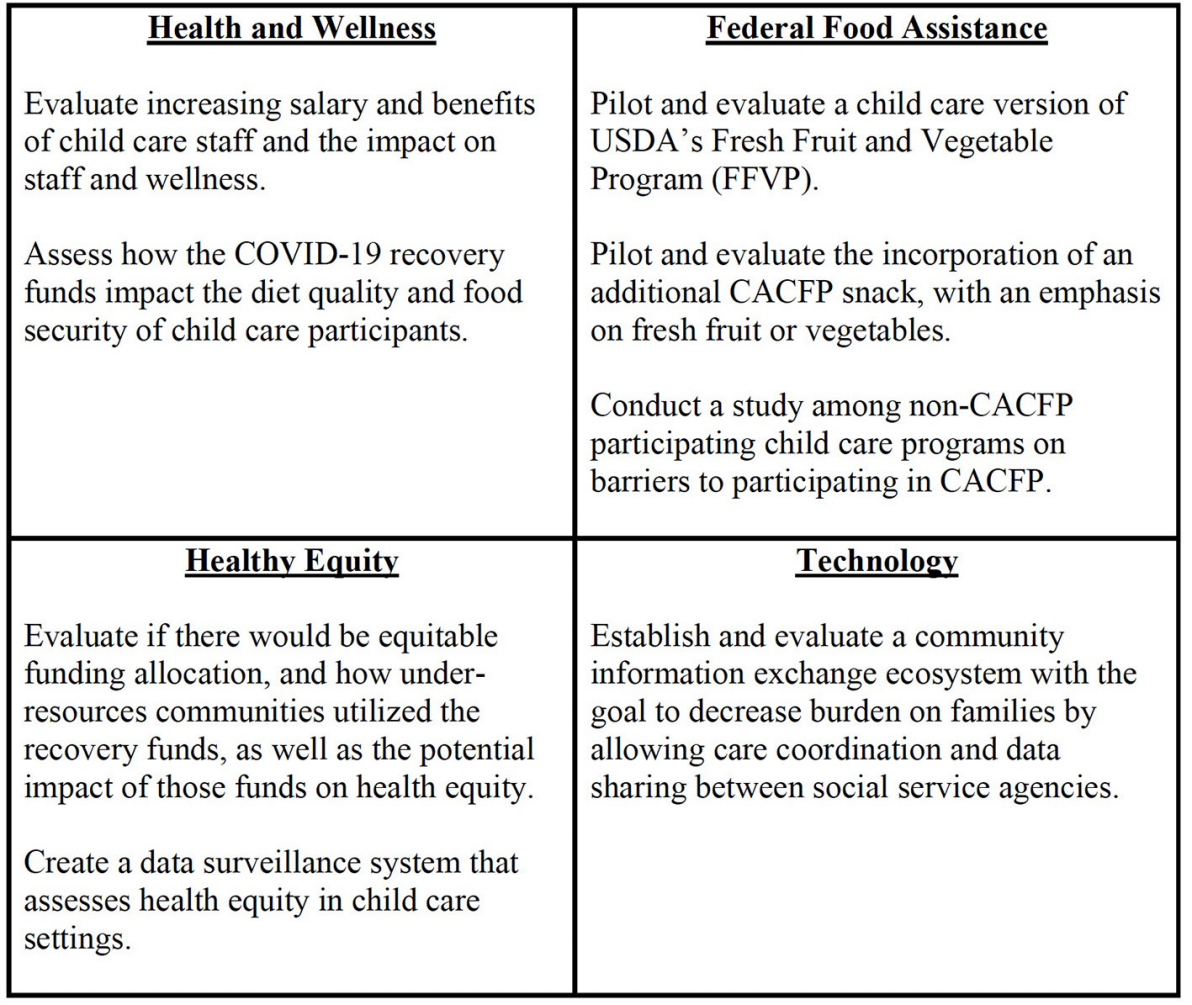
Priority research topics. Suggested research areas and topics that recovery funds could support.

Interviewees, primarily researchers, expressed the importance of evaluating the impacts of COVID-19 recovery funds on nutrition and physical activity in child care programs. Specifically, researchers suggested that recovery funds could be used to support the research areas and projects shown in Figure 1. Researchers indicated that funding opportunities need to include partnerships with community organizations to help ensure sustainability, health equity, and system-level impacts. For instance, community organizations could help with recruitment to ensure study participants are representative of the target population.

A summary of the themes with supplemental quotes on future directions to help recover from COVID-19 is shown in Table 4.

**Table 4:** Key informant themes and quote snapshot: future directions to help recover from COVID-19.

**Theme**	**Example quotes**
Focus on recruiting, retaining & training child care staff	“*[We need] increased salaries for teachers so that we can get teachers back into the centers to provide more care for families. So, we're not dealing with the staffing shortages. We can create a safe environment that encourages families to bring their children back [Practitioner].”*
	“*Teachers that tend to be more interested in movement, tend to promote it more in the class. Teachers that tend to be, and in particular, family child care home folks who are making the meals, who are more interested in nutrition value a lot of nutrition for themselves, tend to make more nutritious meals for the children [Researcher].”*
Form advisory groups of key partners	“*They [need to] have meaningful advisory boards or some type of group that is composed of different sectors of the child care industry in their respective states to ensure that… the plans are rolling out and that they are doing it in the best manner as possible [Practitioner].”*
	“*One of the things for us to do is continue to try to tap into helping states identify for us, where have they been successful in their collaborative partnership development, and then how to help them create connections where they may not have them in place.... we're looking at, who did they say they were partnering with? Who's missing from the table? Who can we connect them to [Federal Representative]?”*
Promote sustainable solutions	“*Think of how you invest in your program rather than just spend money, which is easier said than done, but it is the way to take an opportunity like this, which I think if we all had our druthers, we'd say we'd never wanted this to happen, but it did and now programs have an infusion of capital and they can really put some thought behind it and do some things that forever change their programs [Practitioner].”*
	“*I think, again, you spend money on the things that are going to make a difference, and then you work to educate those who are in decision making authority within your state to help them understand, give them the data, share the outcomes, talk about the benefits [Federal Representative]*.
Prioritize equity	“*I think that's another big divide and risk in these funding streams just reinforcing inequity is maintaining the same systems that limit access to the funds only for programs who know the right person and know the right forms to fill out and places to be. I think that is always an ongoing challenge, whether it's a language barrier, whether it's a capacity barrier, of knowing how to connect to those state entities and funding streams that can help support recovery*
	“*What has happened as a result of seeing the impact of COVID on our population, on our communities, is we know that health is a huge disparity. Now we already knew that…but when it gets highlighted like this in such a big way, it definitely changes the way people think. I know that we will be looking for ways to support our children and families more readily in the area of health and looking at why do those disparities exist? What can we do to mitigate them [Practitioner]?”*

## Discussion

Findings from the interviews demonstrated that the COVID-19 pandemic has exacerbated the major challenges in child care settings that existed pre-pandemic, including child care staff compensation for their role in child development. Specifically, COVID-19 spotlighted the need for staff salary increases along with staff mental health support and sufficient paid time off. This need is consistent with emerging research which showed COVID-19 intensified work inequities, especially regarding wages among ECE educators in child care settings compared to ECE educators in school settings in Virginia (19). In addition, a recent report published by the National Academies of Sciences, Engineering, and Medicine stressed one way to help with COVID-19 recovery is to improve child care staff working conditions by increasing salaries and providing professional development and mental health support opportunities (20). Child care staff also need training in nutrition and physical activity to serve as healthy role models for children in their care.

Resources, such as Nemours Technical Assistance Brief and the Center for the Study of Child Care Employment's public database of compensation strategies, provides guidance regarding how child care settings in eligible states can use child care stabilization grants to strengthen nutrition and physical activity efforts in this setting (21, 22). COVID-19 recovery funds can be used to address staffing issues; however, a more permanent funding mechanism might be needed to sustain changes, such as increasing salaries. If child care programs do increase salaries, they will also need to consider ways to avoid recouping the costs by increasing the cost of care for families. As indicated by interviewees, cost is already a barrier for some families.

COVID-19 also intensified the issue of food insecurity among child care families and staff. Findings from the interviews demonstrated that opportunities need to be in place to support and sustain food security efforts, so that the financial burden is not on the child care providers. Child care partnerships with community organizations, such as food banks and social service programs, could help alleviate food insecurity among child care staff and families. A recent analysis of ECE providers found that child care centers, primarily those participating in CACFP, increased efforts to connect families to food assistance (e.g., “grab and go meals,” food boxes, meal delivery, etc.) through community entities (23).

Prioritizing the SDOH ensures individuals can obtain optimal health and quality-of life outcomes (24). SDOH “are conditions in the places where people live, learn, work, and play that affect a wide range of health risks and outcomes” with early care and education, community and social context, financial resources, and built environment as key areas of SDOH (2). Overall, the findings align with promoting SDOH, including access to nutritious foods and physical activity opportunities and economic stability (2). For instance, the findings highlighted that child care provides an opportunity to reduce food insecurity by serving nutritious foods along with increasing physical activity by incorporating physical activity as part of the child care daily curriculum. In addition, adequate child care staff compensation and benefits can help reduce poverty and increase economic stability. Given SDOH impact health outcomes and health disparities, including obesity and food security, (2, 25) continued programmatic and public health efforts are critical to fostering health and wellbeing in child care settings. For example, the American Academy of Pediatrics, American Public Health Association, and National Resource Center for Health and Safety in Child Care and Early Education compiled an extensive list of evidence-based recommendations for child care providers to help prevent childhood obesity (26) In addition, CDC's Health Equity Resource Toolkit for State Practitioners Addressing Obesity Disparities (27) and Promoting Health Equity: A Resource to Help Communities Address SDOH (28) are resources to help address health disparities associated with SDOH at both the state and local levels.

Researchers could add to this area by conducting impact assessments and pilot studies on child care recovery focused on issues, such as food insecurity and health and wellness. Furthermore, studies could prioritize assessing diet and physical activity behaviors in children and ways to promote these behaviors during challenging times. Since there is a coexistence of food insecurity and obesity, childhood obesity research funding mechanisms could incorporate food insecurity as part of their research agenda (29). Research with observational study designs also is needed that focus on food insecurity and early childhood obesity. A growing body of tools for rigorous evaluation of ‘natural experiments' addressing public health programs and policies provide an avenue for research contributions to SDOH influencing nutrition and physical activity in child care settings (30).

Future research also could investigate ways to improve the health and wellness of child care staff. For instance, formative research could be conducted to identify and test staff health and wellness training curricula. These curricula could have a dual benefit of improving the health status of child care staff while also equipping staff with the knowledge and skills to be healthier role models for children under their care. The Head Start and Early Childhood Learning and Knowledge Center provides resources for promoting staff well-being in child care settings that can be utilized when designing and adapting this type of curricula (31).

Formative research also could be conducted on adding standards related to health and wellness of child care staff and participants to the Quality Rating and Improvement System (QRIS) (32). QRIS is “a systemic approach to assess, improve, and communicate the level of quality in early and school-age care and education programs” (32). States can use CCDF funds to support QRIS activities. Thus, the addition of health and wellness standards to QRIS has the potential for long-term benefits across child care programs nationwide.

This study had a couple limitations to note. First, the study was conducted prior to the surge of the Delta and Omicron SARS-CoV-2 variants, so there could be additional impacts of COVID-19 on child care programs yet to be identified. Second, the results also are based on a convenience sample, and thus, not representative of all federal representatives, practitioners, and researchers involved with child care. In addition, the study did not interview caregivers of children participating in child care. These caregivers could provide valuable insights on the end-user perspective. However, the findings from this study also are supported by several strengths, including successfully recruiting interviewees with an 80 percent response rate. This resulted in conducting multiple interviews with the three different interviewee groups - federal representatives, practitioners, and researchers - ensuring the findings represent feedback from each key audience. Also, the investigators identified several interviewees that resulted in a sufficient respondent pool for interviews to continue until saturation was achieved.

## Conclusion

Findings from this qualitative study highlighted the impacts of COVID-19 on child care settings, including direct effects on child care staff and participating children and families. Interviewees offered potential solutions for recovery related to SDOH, including staff recruitment and retention, food insecurity and improvements to nutrition and physical activity programming. Adjustments made during the pandemic, often through flexibility of federal regulations, allowed new promising practices to emerge, and if made permanent, could continue to benefit child care programs and participants, and ultimately childhood obesity. When considering the use of recovery funds, interviewees emphasized prioritizing long-term and equitable changes. Partnering with key community organizations and gathering input from community members were noted as possible strategies to facilitate the appropriate use of available funds. Future research could be conducted to support these efforts to help ensure changes are evidence-based and sustainable.

## Data Availability Statement

The datasets presented in this article are not readily available because of participant privacy assurances due to the qualitative nature of this study. Requests to access the datasets should be directed to anitto@centerfornutrition.org.

## Ethics Statement

The studies involving human participants were reviewed and approved by University of Nebraska Medical Center Institutional Review Board. Written informed consent for participation was not required for this study in accordance with the national legislation and the institutional requirements.

## Author Contributions

AN, DB, AB, LC, and AY conceptualized the study design and procedures. AN, LC, and AY designed the interview guide. DB and AB reviewed and provided feedback on the interview guide. AN and LC collected data, coded, and analyzed data. AN, DB, AB, SK, LC, and AY interpreted data. AN, SK, and LC drafted the manuscript. DB, AB, and AY reviewed and provided feedback on the manuscript. All authors contributed to the article and approved the submitted version.

## Funding

This study and open access publication fees were funded through a contract awarded by FHI 360 to Gretchen Swanson Center for Nutrition. The contract included funds to produce a report for FHI 360. For more details, visit https://www.nccor.org/projects/impact-of-covid-19-on-child-care-programs-potential-solutions-emerging-opportunities/.

## Conflict of Interest

The authors declare that the research was conducted in the absence of any commercial or financial relationships that could be construed as a potential conflict of interest.

## Publisher's Note

All claims expressed in this article are solely those of the authors and do not necessarily represent those of their affiliated organizations, or those of the publisher, the editors and the reviewers. Any product that may be evaluated in this article, or claim that may be made by its manufacturer, is not guaranteed or endorsed by the publisher.
